# Hydrogen peroxide is a neuronal alarmin that triggers specific RNAs, local translation of Annexin A2, and cytoskeletal remodeling in Schwann cells

**DOI:** 10.1261/rna.064816.117

**Published:** 2018-07

**Authors:** Samuele Negro, Marco Stazi, Marta Marchioretto, Toma Tebaldi, Umberto Rodella, Elisa Duregotti, Volker Gerke, Alessandro Quattrone, Cesare Montecucco, Michela Rigoni, Gabriella Viero

**Affiliations:** 1 Department of Biomedical Sciences, University of Padua, 35131 Padua, Italy; 2 Institute of Biophysics, CNR Unit at Trento, 38123 Povo, Italy; 3 Centre for Integrative Biology, University of Trento, 38123 Povo, Italy; 4 Institute of Medical Biochemistry, University of Münster, 48149 Münster, Germany; 5 CNR Institute of Neuroscience, 35131 Padua, Italy

**Keywords:** Annexin A2, hydrogen peroxide, RNA transport, local protein synthesis, local translation, neuromuscular junction, next-generation sequencing, polysome profiling, Schwann cells, translatome, axonal regeneration

## Abstract

Schwann cells are key players in neuro-regeneration: They sense “alarm” signals released by degenerating nerve terminals and differentiate toward a proregenerative phenotype, with phagocytosis of nerve debris and nerve guidance. At the murine neuromuscular junction, hydrogen peroxide (H_2_O_2_) is a key signal of Schwann cells’ activation in response to a variety of nerve injuries. Here we report that Schwann cells exposed to low doses of H_2_O_2_ rewire the expression of several RNAs at both transcriptional and translational levels. Among the genes positively regulated at both levels, we identified an enriched cluster involved in cytoskeleton remodeling and cell migration, with the Annexin (Anxa) proteins being the most represented family. We show that both Annexin A2 (Anxa2) transcript and protein accumulate at the tips of long pseudopods that Schwann cells extend upon H_2_O_2_ exposure. Interestingly, Schwann cells reply to this signal and to nerve injury by locally translating Anxa2 in pseudopods, and undergo an extensive cytoskeleton remodeling. Our results show that, similarly to neurons, Schwann cells take advantage of local protein synthesis to change shape and move toward damaged axonal terminals to facilitate axonal regeneration.

## INTRODUCTION

Perisynaptic or terminal Schwann cells (PSC) play fundamental roles during reinnervation of the neuromuscular junction (NMJ) following nerve injury. Upon activation by signals emitted by degenerating neurons, they acquire macrophage-like properties which enable them to engulf nerve debris, a preliminary and essential step for anatomical and functional recovery of nerve terminals. PSC guide regenerating motor axons to vacant synaptic sites by extending processes from denervated synapses, whose molecular determinants are poorly known (Son et al. 1996; Duregotti et al. 2015).

The activation of PSC is promoted by an array of signaling molecules that are produced at the site of injury. The nature of only a few of these signals is presently known (Darabid et al. 2014). We recently identified hydrogen peroxide (H_2_O_2_) as a major trigger of NMJ regeneration (Duregotti et al. 2015). H_2_O_2_ is produced by mitochondria of damaged nerve terminals during degeneration caused by the presynaptic neurotoxin α-Latrotoxin (α-LTx), or by complement activation induced by presynaptic-binding autoantibodies (Duregotti et al. 2015; Rodella et al. 2016). Both agents cause a localized and reversible degeneration of motor axon terminals, and their action mimics the cascade of events that leads to nerve terminal degeneration in patients affected by different peripheral neuropathies (Duregotti et al. 2015; Rodella et al. 2016; Rigoni and Montecucco 2017). Neuronal H_2_O_2_ rapidly diffuses across membranes and sets in motion a series of proregenerative responses in PSC, including a profound morphological remodeling (Duregotti et al. 2015), which is likely to result from changes in transcribed and translated mRNAs.

To address this point we profiled the transcriptome and translatome of primary Schwann cells (SC) exposed to a low concentration of H_2_O_2_. We obtained the complete list of transcripts differentially regulated upon treatment at both transcriptional and translational levels, many of them falling in functional categories related to cytoskeleton remodeling and motility, including the Annexin (Anxa) family. We validated our high-throughput data set and investigated the role of Anxa2 in injury-induced SC plasticity, providing evidence that SC are capable of triggering Anxa2 local protein synthesis in pseudopods. These findings unravel a biological role for Anxa2 as an important player in the remarkable change of shape of SC during nerve terminal regeneration, relevant for to the entire process of NMJ regeneration.

## RESULTS

### Transcriptome and translatome profiles of primary SC reveal gene expression rewiring upon H_2_O_2_ exposure and coordinated up-regulation of Annexin family members

**FIGURE 1.**
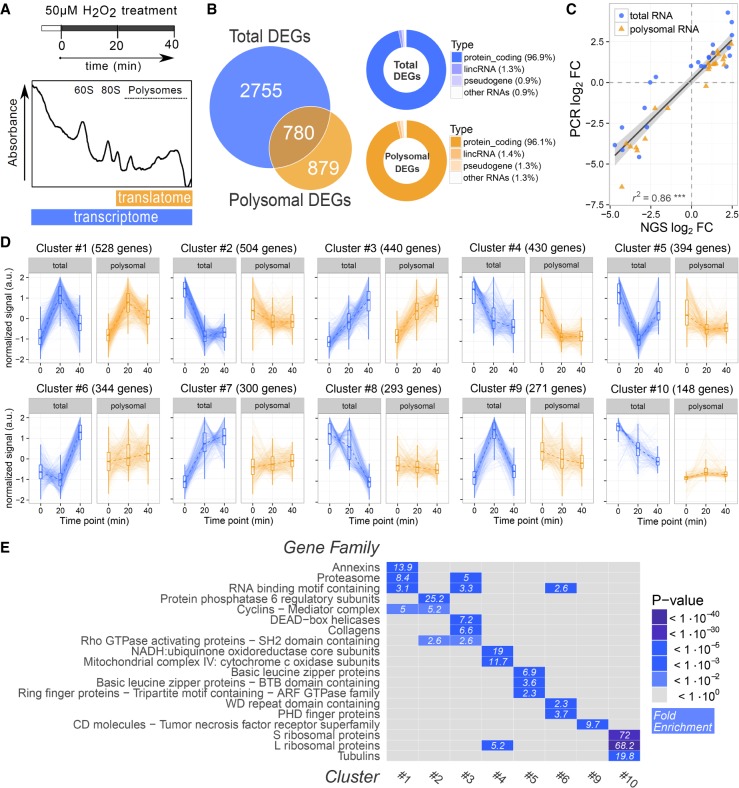
RNA-seq and POL-seq of SC upon H_2_O_2_ treatment reveal specific coregulation of gene families. (*A*) Scheme of polysomal profiling of primary SC isolated from rat sciatic nerves and treated with 50 µM H_2_O_2_ for 20 and 40 min. (*Bottom*) Representative sucrose gradient profile highlighting the fractions used for the preparation of polysomal and total RNA libraries, respectively. (*B, left*) Venn diagram showing the number of differentially expressed genes (DEGs) specifically or commonly identified in transcriptome and translatome profilings. (*Right*) Donut charts representing the RNA class composition of total and polysomal DEGs. The majority (96%) of identified DEGs are protein coding transcripts. (*C*) Scatter plot comparing NGS and qPCR results on a set of 11 differentially expressed genes upon H_2_O_2_ treatment. The significant coefficient of determination, as calculated by linear regression, demonstrates the high level of agreement between the two techniques. Both total RNA (circle) and polysomal RNA (triangle) data are shown. (*D*) Cluster analysis of DEGs. Clusters were identified based on expression correlation values using the affinity propagation method. Clusters were ranked by decreasing size, and the ten clusters with more than 100 genes are displayed. Each plot represents the total and polysomal expression trajectories of the single genes belonging to the cluster, together with the cluster median (dotted line) and summary statistics (boxplot). (*E*) Heat map displaying gene family enrichment analysis on cluster of DEGs. For each cluster shown in panel *D*, the *top* enriched gene families are shown. Significant enrichments are displayed in blue shades, with the corresponding fold enrichment value indicated in each tile.

Our finding that neuronal-derived H_2_O_2_ triggers motor axon terminal regeneration by activating PSC (Duregotti et al. 2015) prompted us to study the underlying changes in gene expression to identify cellular and molecular mechanisms involved in the process. To this aim we took advantage of polysomal profiling, a classical approach to study translation (King and Gerber 2016), and compared the changes at transcriptional and translational levels occurring in response to H_2_O_2_. Primary SC were exposed to the stimulus (50 µM H_2_O_2_ for 20 and 40 min) and harvested. Polysomes were separated from ribosomal subunits, nontranslating ribonucleoparticles and ribosomes on miniaturized sucrose density gradients, and their sedimentation profiles were measured by absorbance at 254 nm (Fig. 1A). The percentage of ribosomes engaged on polysomes was calculated considering the ratio between the absorbance of polysomes and the total absorbance of ribosomes of the cells (nontranslating monosomes (80S) plus polysomes (Supplemental Fig. S1a). The comparison of this value between different conditions gives an estimation of global variations in translation (Bernabò et al. 2017). We observed a trend of slight translational depression, suggestive of translational reorganization. Since H_2_O_2_ exposure is known to induce the activation of multiple pathways involved in translational control (Grant 2011; Piccirillo et al. 2014), we monitored the possible activation of ERK, PERK, and mTORC pathways. As expected, we found that these pathways are activated early after H_2_O_2_ exposure (Supplemental Fig. S1b,c).

To detail the whole set of changes occurring in SC at transcriptional and translational levels, cytosolic RNA (RNA-seq) and polysomal RNA (POL-seq) were purified and sequenced by next-generation sequencing (NGS). Differential expression analysis identified a large number of genes with significant transcriptome and translatome changes (3535 and 1659, respectively), with 780 overlapping genes (Fig. 1B). These numbers suggest that most of transcriptome variations do not extend to the translatome compartment, appointing polysome profiling as a valid technique to filter out expression changes that are consistent along the whole expression axis (Tebaldi et al. 2012). Most of the differentially expressed genes (DEGs) are protein coding (96%), with small percentages of noncoding transcripts (Fig. 1B, right panel). A set of 11 DEGs in both total and polysomal sequencing were used to validate NGS data (Fig. 1C). We compared the fold change values obtained by qPCR with those obtained by NGS for both levels, showing a high concordance among the two techniques (coefficient of determination = 0.83, *P* < 0.001).

**FIGURE 2.**
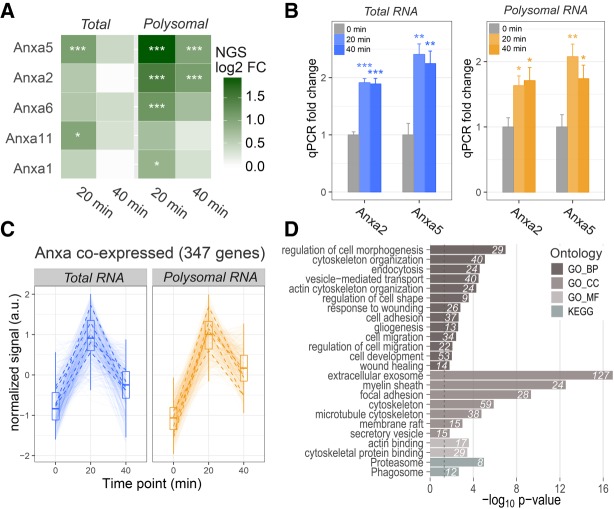
Annexins are coordinately early up-regulated in response to H_2_O_2_. (*A*) Heatmap of Anxa gene expression changes upon H_2_O_2_ treatment, determined from RNA-seq and POL-seq. Log_2_ fold changes compared to untreated cells are displayed, and significant changes are labeled ([*] *P* < 0.05, [***] *P* < 0.001). Anxa members are ordered according to their average expression fold change. (*B*) qPCR fold changes of Anxa2 and Anxa5 in total and polysomal RNA fractions after exposure to H_2_O_2_ for 20 and 40 min. For each transcript, the mean value ± SE (four biological replicates and two to three technical replicates) is shown. Significant changes between control and H_2_O_2_-treated SC were measured with *t*-test ([*] *P* < 0.05, [**] *P* < 0.01, [***] *P* < 0.001). (*C*) Total and polysomal RNA expression trajectories of 347 genes significantly coregulated with Anxa proteins. Expression summary statistics are displayed as box-whisker plots. Trajectories of the five Anxa mRNAs are highlighted with black dashed lines. (*D*) Barplots displaying enriched functional terms and pathways among genes coregulated with Anxa transcripts upon H_2_O_2_ treatment (GO: Gene Ontology, BP: Biological Process, CC: Cellular Component, MF: Molecular Function). Bar lengths are proportional to enrichment *P*-values. The number of genes falling into each category is displayed next to each bar.

To identify groups of coexpressed genes upon H_2_O_2_ treatment, DEGs were clustered by the affinity propagation approach (Frey and Dueck 2007), based on expression correlation values as distance measurements. Ten clusters of coregulated genes with size >100 genes were identified, showing distinctive expression trajectories (Fig. 1D). For example, Cluster #1 comprises “early responding” genes, with a peak of up-regulation at 20 min after H_2_O_2_ exposure, while Cluster #3 includes genes with constant increase in expression during treatment. Genes belonging to clusters #2, #4, and #5 are down-regulated following the treatment. Notably, the first five clusters (top row in Fig. 1D) comprise genes with a concordant response to H_2_O_2_ at both transcriptome and translatome levels, whereas the others mostly include genes with transcriptome variations not accompanied by parallel translatome changes. The full data set of gene expression changes belonging to each cluster is available in Supplemental Table S3. To better characterize the composition of coregulated groups of genes, we performed gene family enrichment analysis of each cluster. This analysis reveals the presence of specific gene families in the majority of clusters (Fig. 1E). Notably, the Annexin (Anxa) family was identified as the most enriched among early responding up-regulated transcripts (Cluster #1, Fig. 1D, first row), with five members early up-regulated at both total and polysomal level upon H_2_O_2_ treatment: Anxa5, Anxa2, Anxa6, Anxa11, and Anxa1 in decreasing order of fold change (Fig. 2A). Up-regulation of Anxa5 and Anxa2 was confirmed by qPCR (Fig. 2B). By performing a correlation analysis we filtered out a set of 347 genes significantly coexpressed with Anxa members, with an “early up-regulation” trend during H_2_O_2_ response (Fig. 2C). Gene Ontology enrichment analysis of this population revealed as top over-represented biological processes “endocytosis,” “cytoskeleton organization,” “regulation of cell shape,” “cell adhesion,” “cell migration,” “gliogenesis,” “wound healing,” with “extracellular exosome,” “myelin sheath,” “cytoskeleton,” and “membrane raft” being the most represented cellular compartments (Fig. 2D). This finding is particularly significant as SC modify their cell shape following motor axon terminal damage, becoming elongated and highly mobile. These changes involve a marked reorganization of the cell cytoskeleton. In this respect, we first studied the intracellular distribution of Anxa5 and Anxa2, the most up-regulated Anxas, before and after treatment of SC with H_2_O_2_.

### Cellular redistribution of Anxa2 in SC upon H_2_O_2_ treatment

**FIGURE 3.**
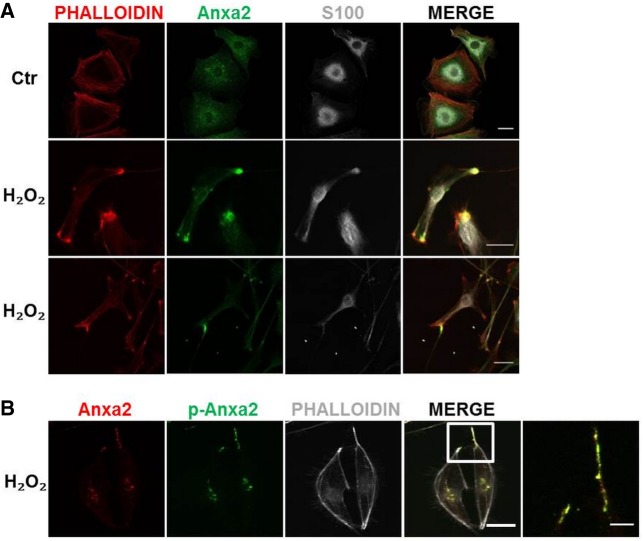
Anxa2 cellular localization and phosphorylation status in primary SC upon H_2_O_2_ stimulation. (*A*) Anxa2 (green) accumulates in phalloidin-positive (red) foci in primary SC (S100 positive, white) upon exposure to 50 µM H_2_O_2_ for 10 min. Scale bars, 20 µm. (*B*) Tyr23-phosphorylated (green) Anxa2 (red) accumulates at cell periphery upon exposure of primary SC (S100-positive, white) to 50 µM H_2_O_2_ for 10 min. Scale bars, 20 µm.

While Anxa5 displays a clear and almost exclusive nuclear localization in both resting and H_2_O_2_-activated SC (S100 positive, Supplemental Fig. S2), Anxa2 shows a diffuse and punctuated cytoplasmic distribution (Fig. 3A). H_2_O_2_ treatment induces an impressive change in cell shape: SC become bipolar and start extending long pseudopods. Remarkably, Anxa2 accumulates at the leading edges of these processes together with actin (Fig. 3A). This is in line with the recently reported role of Anxa2 in actin nucleation by local translation of mRNAs (Liao et al. 2015).

As phosphorylation of Tyr23 of Anxa2 favors actin dynamics and migration (Rescher et al. 2008), we assessed Anxa2 Tyr23 phosphorylation after H_2_O_2_ treatment with a specific antibody. We found that phosphorylated Anxa2 concentrates at the leading edges of the elongated SC (Fig. 3B), suggesting that it may be involved in mRNA transport and translation of actin to reorganize the cytoskeleton.

### SC processes polarize in response to H_2_O_2_ and to neuronal injury

**FIGURE 4.**
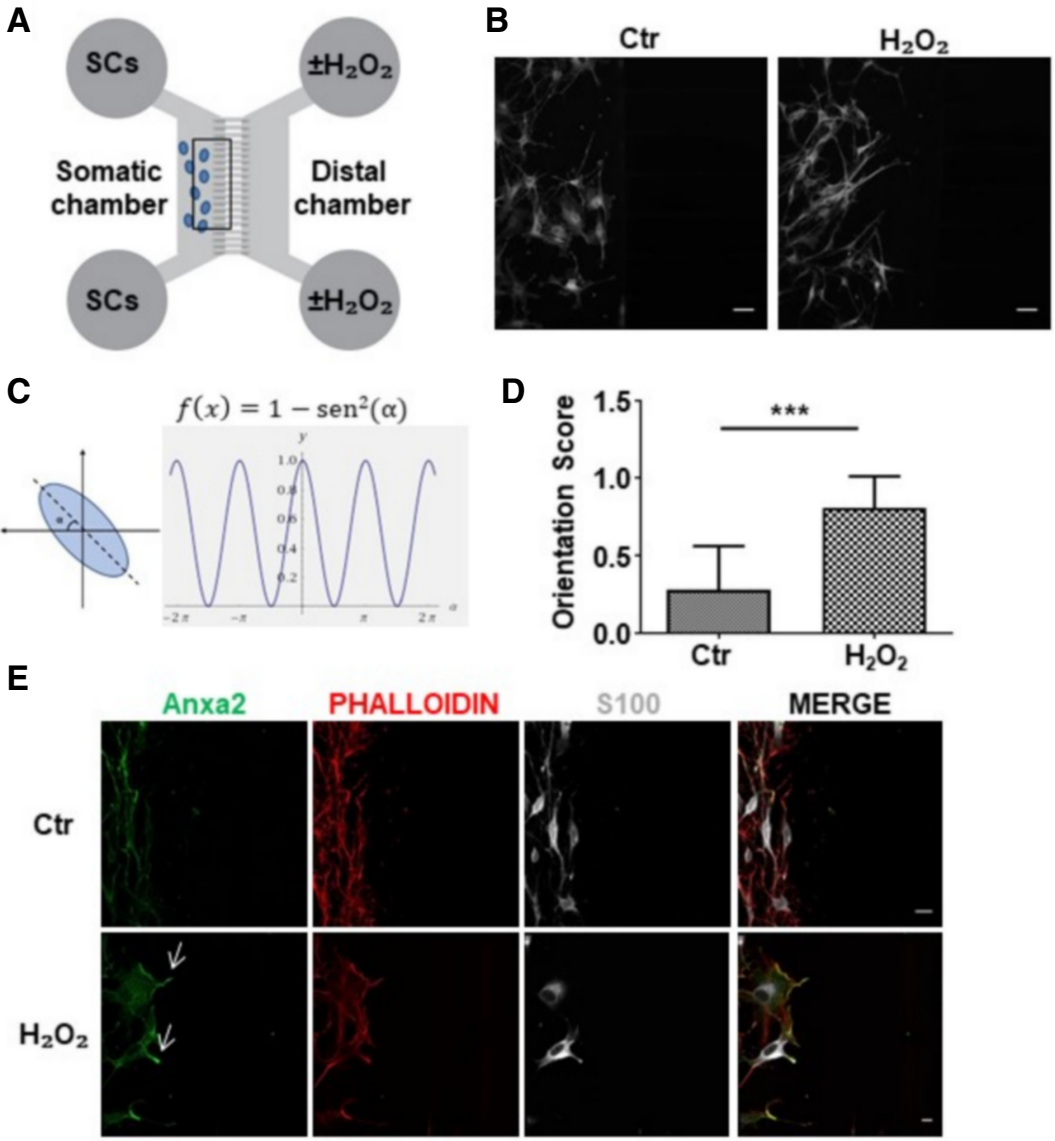
H_2_O_2_ triggers polarization of primary SC cultured in microfluidic devices. (*A*) Scheme of microfluidic devices used in the study. (*B*) SC (S100 positive, white) plated in the somatic chamber are exposed to H_2_O_2_ added to the distal one (50 µM for 1 h, *right* panel). SC processes orientate toward the opposite compartment. Scale bars, 50 µm. (*C*,*D*) Orientation score determination. (***) *P* < 0.001. (*E*) Anxa2 (green) accumulates close to the grooves' entrance upon H_2_O_2_ stimulation. SC are identified by S100 staining (white); phalloidin is in red. Scale bars, 20 µm.

The remarkable shape change triggered by H_2_O_2_ in SC was further studied using compartmentalized cultures. H_2_O_2,_ applied to the distal chamber of microfluidic devices, with SC plated in the somatic one (Fig. 4A), clearly behaves as a chemoattractant for SC. In fact, SC extend long processes along the concentration gradient generated across the grooves (Fig. 4B). The orientation score (OS), defined as in Figure 4C, allowed us to quantify this effect (Fig. 4D), which is statistically significant and concomitant with Anxa2 colocalization with phalloidin (Fig. 4E). Interestingly, Anxa2 accumulates in close proximity to the entry of the microgrooves (arrows in Fig. 4E).

**FIGURE 5.**
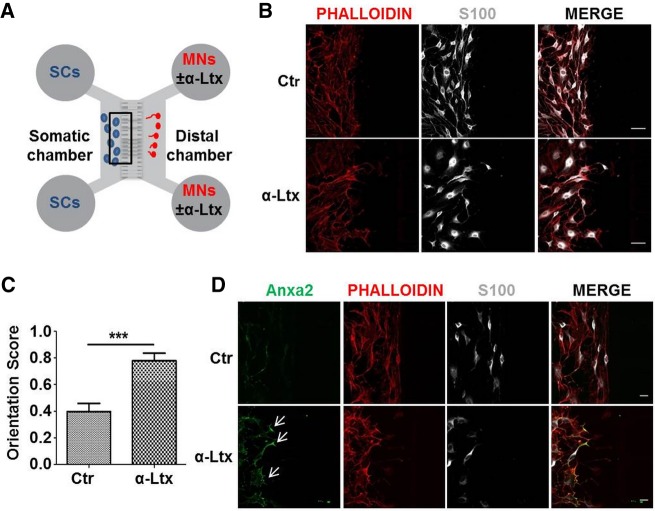
Neuronal degeneration triggers SC polarization. (*A*) Scheme of microfluidic devices used, with SC plated in the proximal compartment and MN in the distal one. (*B*) Upon MN exposure to α-LTx (1 nM for 1 h), SC processes (phalloidin-positive, red) orientate toward the microfluidic grooves connecting the two chambers. (*C*) Orientation score quantification. (***) *P* < 0.001. (*D*) Intoxication leads to Anxa2 (green) accumulation close to the grooves’ entrance (arrows). SC are identified by S100 staining (white). Scale bars, 50 µm.

We then studied SC remodeling and Anxa2 localization upon nerve terminal injury in motor neurons (MN). We used an in vitro model of reversible neuronal degeneration, consisting in exposing primary motor neurons (MN) to the spider presynaptic neurotoxin α-LTx (Rigoni and Montecucco 2017). The intoxication leads to the release by degenerating neurons of a number of “alarmins” (among them H_2_O_2_), which trigger SC phagocytosis in vitro and in vivo (Supplemental Fig. S3; Duregotti et al. 2015). When MN grown in the distal chamber of microfluidic chambers are exposed to α-LTx (Fig. 5A), SC processes orientate toward the distal compartment, indicating that they “sense” neuronal alarmins diffusing through the grooves (Fig. 5B). Quantification of the orientation score is shown in Figure 5C. Also in this coculture experiment, Anxa2 signal accumulates close to the channel entrance (Fig. 5D, arrows), supporting a role of Anxa2 in the spatial reorganization of the actomyosin cytoskeleton.

### Local protein translation in SC peripheral processes

**FIGURE 6.**
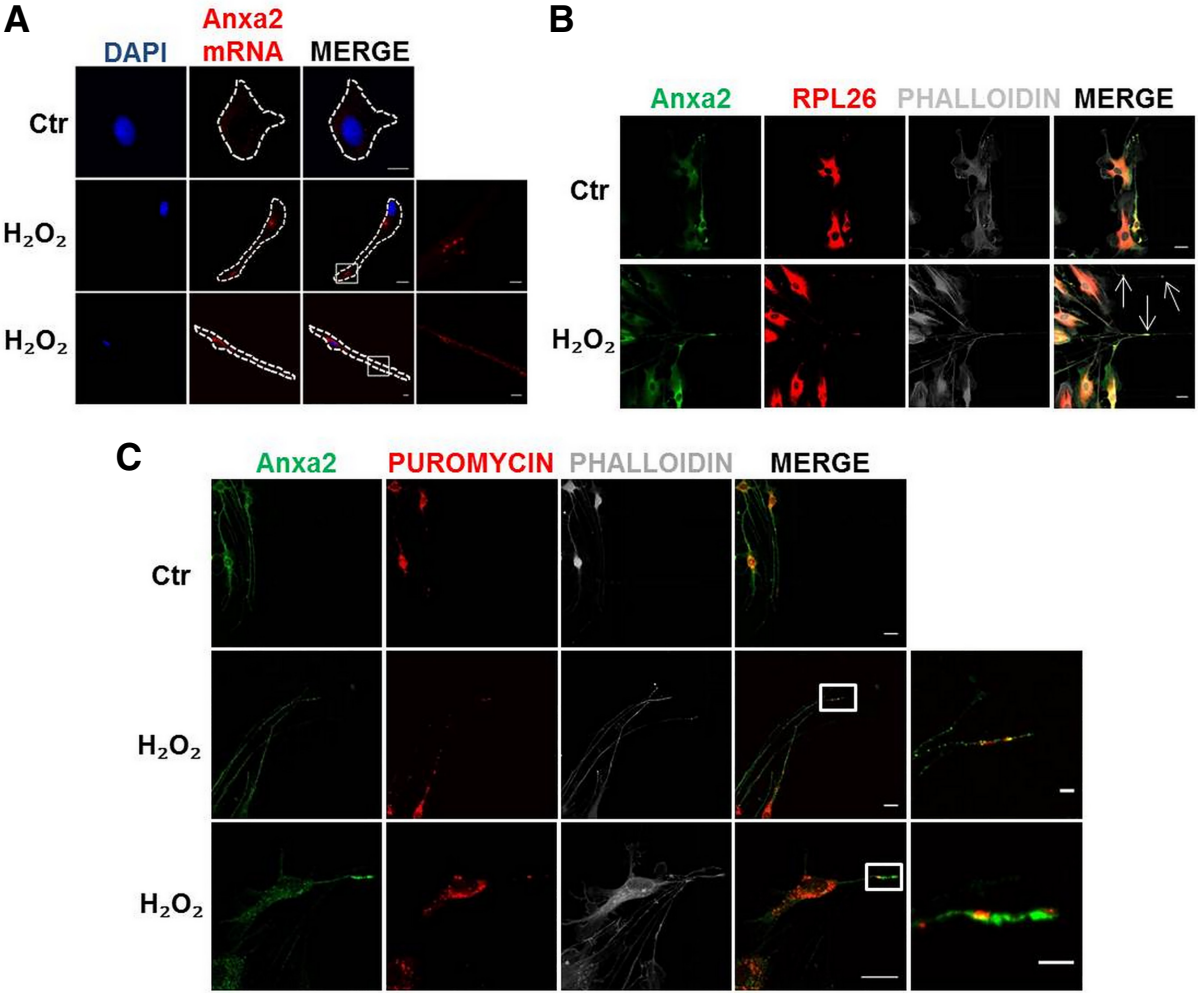
SC local translation. (*A*) In situ hybridization shows Anxa2 mRNA spots along SC processes. DAPI stains cell nuclei. Scale bars, 10 µm (magnification 2 µm). (*B*) Partial colocalization of RPL-26 (red) and Anxa2 (green) in SC processes growing toward the distal compartment of microfluidic devices following H_2_O_2_ concentration gradient (SC plated in the proximal chamber, H_2_O_2_ added to the distal one, 50 µM for 1 h). Phalloidin is in white. Scale bars, 20 µm. (*C*) Puromycin-positive spots (red) are detectable in SC cytoplasm. Upon H_2_O_2_ addition to the distal compartment of microfluidic devices (50 µM for 1 h), some red puncta are detectable along cell processes growing toward the distal chamber (SC in the proximal one). Puromycin-labeled puncta partially colocalize with Anxa2-positive spots (green) in SC processes. Phalloidin staining is in white. Scale bars, 20 µm (magnification 5 µm).

On the basis of these results, we wondered whether local protein synthesis of Anxa2 was responsible for Anxa2 protein accumulation in SC in correspondence to actin foci at the cell periphery. Sequence analysis of the 3′UTR of Anxa transcripts revealed that Anxa2 transcript contains a sequence motif consistent with the Quaking Response Element (QRE), not present in other Anxa transcripts. Since the QRE was recently found enriched in transcripts locally translated in PAPs (peripheral astrocytic processes) (Sakers et al. 2017), we hypothesized that Anxa2 mRNA might be transported at the cell borders to be locally translated. To test this possibility, we performed in situ hybridization to determine the cellular distribution of Anxa2 mRNA, and found that Anxa2 mRNA is transported in SC processes in response to H_2_O_2_ stimuli (Fig. 6A). We then checked for the possible colocalization of Anxa2 with the translation machinery. To this aim we used a marker for ribosomes, the ribosomal protein RPL26, which enables a localization of ribosomes and polysomes using immunofluorescence techniques (Viero et al. 2015). In microfluidic devices RPL26-positive spots localize in SC processes only after the formation of the H_2_O_2_ gradient along the microgrooves (Fig. 6B). Importantly, RPL26 frequently colocalizes with Anxa2 signal (Fig. 6B, arrows), suggesting that Anxa2 is likely translated locally. Finally, to further test whether these foci are sites of active local translation for Anxa2, we used immunofluorescence of Anxa2 protein combined to the puromycilation assay. This latter technique uses puromycin, a tRNA-structural analog, to identify foci of active translation by incorporating puromycin into translating ribosomes and binding the growing peptides within ribosomes (David et al. 2012). Using an antibody against puromycin, we detected puromycin-positive spots also along SC processes, indicative of active local protein synthesis (Fig. 6C). Importantly, a fraction of these peripheral foci overlapped with Anxa2-positive puncta. Overall these results provide strong evidence that SC locally translate Anxa2 upon H_2_O_2_ stimulus.

## DISCUSSION

We have defined here the transcriptome and translatome profiles of primary SC exposed to H_2_O_2_, a major alarm signal released by degenerating nerve terminals following acute damage. H_2_O_2_ plays a major role in NMJ recovery of function in a model of reversible peripheral nerve injury caused by animal presynaptic neurotoxins, by inducing PSC activation (Duregotti et al. 2015).

Both total and polysomal RNAs from control and H_2_O_2_-treated SC were sequenced: The acute exposure of primary SC to micromolar H_2_O_2_ concentrations triggers changes in the expression of a large number of genes. By clustering gene expression trajectories, we identified large sets of transcripts whose changes are coordinated at both transcriptional and translational levels in response to the treatment. Among those clusters positively regulated at both levels, the largest in size is enriched in members of the Anxa protein family. Additionally, transcripts associated to cytoskeleton remodeling and motility are also positively up-regulated at transcriptional and translational levels, suggesting that both Anxa proteins and transcripts are likely involved in cytoskeleton remodeling of SC induced by H_2_O_2_ treatment.

Anxa family members bind phospholipids in a Ca^2+^-dependent manner, a property that links them to membrane-related events, such as cytoskeleton remodeling, providing a link between Ca^2+^ signaling and membrane functions. By forming reversible networks on the membrane surface, Anxa proteins act as organizers of membrane domains in a variety of cells and cellular processes (for review, see Gerke et al. 2005). These include changes in cell shape, e.g., the acquisition of an apico-basolateral polarity in epithelial cells that requires a phosphoinositide-mediated recruitment of Anxa2 to the apical membrane domain (Martin-Belmonte et al. 2007), a role of Anxa1 in the formation of membrane contact sites between endosomes and the ER (Eden et al. 2016), and a function of several annexins in the Ca^2+^-dependent resealing of plasma membrane wounds (McNeil et al. 2006; Babiychuk et al. 2009; Boye et al. 2017). As PSC undergo profound morphological and functional changes upon neuronal damage or paralysis (Duregotti et al. 2015), including the acquisition of phagocytic behavior, the involvement of Anxa members in SC plasticity and remodeling upon injury appears very likely. In this regard, (Clements et al. 2017) carried out transcriptional profiling of in vivo SC from intact nerves and from bridge and distal stumps of transected nerves. By using their published RNA-seq data set, we could confirm that both Anxa2 and Anxa5 are significantly up-regulated in bridge and distal regions of transected nerves, with an expression peak at 4 d after injury (Supplemental Fig. S4). Moreover, Arthur-Farraj et al. (2017) reported Anxa2 as significantly up-regulated from RNA-seq analysis of distal nerve stumps 7 d after sciatic nerve cut, compared to the uncut nerve. We therefore investigated the effects of H_2_O_2_ and neurodegeneration on SC plasticity, concentrating on Anxa5 and Anxa2, whose transcripts are the most up-regulated among the Anxa family members at both transcriptional and translational levels.

The Anxa5 protein was detected in the nucleus of both resting and H_2_O_2_-treated SC, in agreement with previous reports on a nuclear localization of both Anxa1 and Anxa5 (Raynal et al. 1992; Sun et al. 1992; Koster et al. 1993). This result suggests a possible involvement of Anxa5 in the regulation of nuclear function, requiring further investigation which is beyond the scope of the present work.

In cultured SC, Anxa2 protein is cytosolic, and its expression and distribution change upon H_2_O_2_ exposure in parallel to a remarkable cell elongation. In treated samples, Anxa2 accumulates in phalloidin-positive foci at the leading edge of long processes. When H_2_O_2_ is added to one chamber of microfluidic devices, this signaling molecule acts as a chemoattractant for SC plated in the opposite compartment, causing cellular polarization and extension of long processes that enter the grooves connecting the two chambers. Interestingly, during these remodeling events Anxa2 accumulates in close proximity to the grooves' entrance, pointing toward the chemoattractant source. These results suggest that Anxa2 may be involved in the initial organization of the long SC processes by participating in the polarized rearrangement of the actomyosin cytoskeleton. Neuronal mediators trigger Anxa2 relocalization, as suggested by the evidence that Anxa2 accumulates in the leading edges of projections that SC extend when they receive juxtacrine signals from axons (Poitelon et al. 2015).

Anxa2 undergoes several post-translational modifications (phosphorylations at different sites, acetylation, sumoylation, and others), which are believed to account for the very different functions of the protein and its different ligands (F-actin, mRNA, S100A10, plasminogen, plasmin) (Glenney and Tack 1985; Johnsson et al. 1988; Raynal and Pollard 1994; Gerke et al. 2005; Hollås et al. 2006; Caron et al. 2015). One of the most important post-translational modifications is the phosphorylation of Tyr23, which favors actin dynamics and is required for Anxa2 binding to endosomes and to multivesicular bodies, leading to Anxa2 internalization in exosomes for subsequent extracellular delivery. Oxidative stress also induces up-regulation and phosphorylation of the protein. The levels of Anxa2 and its Tyr23-phosphorylated form increase in several cancers, and the protein is involved in malignant cell transformation, metastasis, and angiogenesis (Mussunoor and Murray 2008). All these processes require cytoskeletal rearrangements and result in morphological changes, and can possibly be linked to the ability of Anxa2 to bind lipids and actin. Accordingly, we found here that Anxa2 accumulated in SC foci is Tyr23-phosphorylated.

What remains to be determined is whether Anxa2 is required to initiate or sustain the actin cytoskeleton changes in SC after H_2_O_2_ exposure, and which mediators of Anxa2 function are involved. Previous work showed that the small GTPase protein CDC42 participates in the interaction between apical membrane phosphatidylinositol (4,5)-bisphosphate (PtdIns(4,5)P2) microdomains and the scaffolding protein Anxa2 (Martin-Belmonte et al. 2007). We have attempted to inhibit CDC42 with the specific inhibitor Casin, but this compound resulted to be very toxic to cultured SC (results not shown). The involvement of small GTPases in the process is also suggested by the increased expression of mRNAs encoding for proteins containing SH2 domain activating Rho GTPases (Fig. 1E).

In response to environmental signals, cells are able to adjust global and local proteomes by modifying subcellular mRNA localization and controlling local protein synthesis. In neurons, local protein synthesis takes place in dendrites and axons during events of synaptic plasticity (Tennyson 1970; Bunge 1973; Steward and Ribak 1986; Bassell et al. 1998; Koenig et al. 2000; Li et al. 2005; Kun et al. 2007; Jung et al. 2012; Shigeoka et al. 2016). Neurons are thought to require localized translation also to compensate for the great length of their dendrites and axons. Translational events or local adaptation of proteomes in glial cells are much less characterized (Love and Shah 2015; Sakers et al. 2017). It is likely that astrocytes contacting multiple synapses may use local translation to allow them to respond to or modulate the activity of specific synapses.

Accordingly, by labeling ribosomes and cellular foci of active protein synthesis in situ, we confirmed the induction of local translation events in SC as a response to H_2_O_2_ chemoattractant. Several puromycin positive-spots are detectable along cell processes that form upon H_2_O_2_ stimulation, and the ribosomal protein RPL26 largely colocalizes with Anxa2 along cell pseudopods. Moreover, Anxa2 mRNAs are also detected by FISH, showing that Anxa2 is indeed synthesized at the leading edges of SC projections. Anxa2 has been reported as a multifunctional RNA binding protein regulating translation of certain mRNAs on cytoskeleton-bound polysomes (Vedeler and Hollås 2000; Hollås et al. 2006; Aukrust et al. 2007). Therefore, the increased local translation of Anxa2 at the leading edges of Schwann cells projections supports its role in the spatial reorganization of the actomyosin cytoskeleton.

These results are in line with accumulating evidences that cells exposed to H_2_O_2_ undergo a complex reprogramming of gene expression, including a global rewiring of translation (Shenton et al. 2006; Grant 2011; Gerashchenko et al. 2012). This includes the post-translational control of general factors of translation (Grant 2011), that are downstream targets of well-known pathways controlling protein synthesis (Piccirillo et al. 2014). Although a primary response to H_2_O_2_ is typically a bulk reduction in protein synthesis, translation of specific mRNAs also occurs (Shenton et al. 2006; Gerashchenko et al. 2012). This event includes the synthesis of proteins that are part of the H_2_O_2_ signaling response (Shenton et al. 2006; Gerashchenko et al. 2012). Hence, translation regulatory mechanisms are activated by H_2_O_2_ sensing, and conveniently reprogram protein synthesis locally to remodel cell shape. Accordingly, local protein synthesis plays an important role in cellular migration and adhesion (Bergeman et al. 2016), suggesting that spatial regulation of protein production is a strategy for the cell to conveniently respond to environmental stimuli.

We have shown here that primary SC respond to H_2_O_2_ by inducing the selective rewiring of transcription and translation programs, that is likely of functional relevance for the morphological remodeling required for successful regeneration. Our findings suggest that gene expression rewiring and local protein synthesis induced by H_2_O_2_ support SC activation during the regeneration process. Anxa2 appears to participate to the initial stages of cytoskeletal remodeling that prepare phagocytosis.

## MATERIALS AND METHODS

### Toxins and antibodies

Purified α-LTx was purchased from Alomone. The purity of the toxin was checked by SDS-PAGE and its neurotoxicity by ex vivo mouse nerve-hemidiaphragm preparations as previously described (Rigoni et al. 2005). Unless otherwise stated, all reagents were purchased from Sigma.

The following primary antibodies and working dilutions were used. For immunofluorescence: anti-β_3_ tubulin (Synaptic Systems, 1:200), anti-Anxa2 (Abcam, 1:200), anti-Anxa5 (Abcam, 1:200), anti-phospho-Anxa2 (Tyr23) (S. Cruz, 1:50), anti-SNAP25 (SMI81) (Covance, 1:200), anti-S100 (Dako, 1:200), anti-RPL26 (Abcam, 1:100), and anti-puromycin (Millipore, 1:5000) were used. Phallodin-555 (Sigma, 1:200) was used to stain actin filaments. Alexa-conjugated secondary antibodies were purchased from Thermo Fisher Scientific (1:200).

For western blots, anti-phospho ERK1/2 (Cell Signaling, 1:1000), anti-phospho 4E-BP (Abcam, 1:1000), anti-phospho eIF2α (S.Cruz, 1:1000), anti-Hsp90 (Synaptic Systems, 1:10000), and secondary antibodies conjugated to HRP (1:2000, Thermo Fisher Scientific) were used.

### Animals

C57BL/6 mice expressing cytosolic GFP under the *plp* promoter (Mallon et al. 2002) were kindly provided by Prof. W.B. Macklin (Aurora, Colorado) and Prof. T. Misgeld (Munchen, Germany). All experimental procedures involving animals and their care were carried out in accordance with National laws and policies (D.L. n. 26, March 14, 2014) and with the guidelines established by the European Community Council Directive (2010/63/UE), and approved by the local authority veterinary services.

### Primary cell cultures

Primary cultures of spinal cord motor neurons (MN), SC and the relative cocultures were prepared as described previously (Rigoni et al. 2008; Duregotti et al. 2015).

### Polysomal extraction

Polysomal lysates were obtained from SC (6 × 10^5^ cells/well seeded in six well plates till 60%–70% confluence) as described in Viero et al. (2015). After a 3 min treatment with 10 µg/mL cycloheximide at 37°C to trap the ribosomes on the mRNA, cell lysates were collected in control condition or after exposure to 50 µM H_2_O_2_ for 20 and 40 min. Lysates were then centrifuged at 14,000*g* for 5 min at 4°C to allow the sedimentation of cellular debris, nuclei and mitochondria. The supernatants were kept on ice for 20 min and then transferred onto a 15%–50% linear sucrose gradient or stored at −80°C. The 15%–50% sucrose gradient was produced in 2.2 mL ultracentrifuge tubes. Sucrose solutions were prepared in 100 mM NaCl, 10 mM MgCl_2_, 30 mM Tris–HCl, pH 7.5. Samples were centrifuged in a Beckman Optima TL Ultracentrifuge for 100 min at 180,000*g* at 4°C using a Beckman TLS-55 swinging rotor. To collect sucrose fractions and measure the absorbance at 254 nm, a Teledyne Isco model 160 gradient analyzer equipped with a UA-6 UV/VIS detector was used. The resulting 250 µL fractions were directly used to extract total cytoplasmic or polysomal RNA according to Bernabò et al. (2017).

### RNA extraction, libraries preparations, and sequencing

The total cytosolic RNA (pool of all sucrose fractions) and the polysomal RNA (collection of fractions corresponding to polysome peaks) were purified using the Direct-zol RNA Purification Kit (Zymo Research).

The libraries for NGS were obtained using the Ovation Single Cell RNA-seq System (Nugen) according to manufacturer's protocol. The qualitative and quantitative controls of the libraries were performed with a High Sensitivity DNA Chip (Agilent Technologies) before sequencing. Total cytoplasmic and polysomal RNA sequencing (RNA-seq and POL-seq, respectively) were performed with Illumina HiSeq 2000 by the CIBIO NGS facility of the University of Trento. The experiments were performed in triplicate.

### NGS data analysis

Barcoded libraries were pooled and sequenced in three HiSeq 2000 lanes. Fastq files were quality checked with FastQC. Reads (100 bp in length) were aligned to the rat genome (genome assembly Rnor_6.0) with Tophat (version 2.0.14), using the Ensembl transcript annotation (Ensembl release 81, *Rattus norvegicus*) as transcriptome guide. All programs were used with default settings unless otherwise specified. Mapped reads (∼80% of total reads, see Supplemental Table S1) were subsequently assembled into transcripts guided by reference annotation (Ensembl release 81, *Rattus norvegicus*) with Cufflinks (version 2.2.1). Expression levels were quantified by Cufflinks with normalized FPKM (fragments per kilobase of exon per million mapped fragments). Differentially expressed genes and transcripts were detected with CuffDiff with a double threshold on the log_2_ fold change (absolute value >0.75) and the correspondent statistical significance (*P* < 0.05). Cluster analysis of DEGs was performed with the affinity propagation clustering method (Frey and Dueck 2007), implemented in the APCluster Bioconductor package. Kendall correlation between scaled expression values was used to generate the similarity matrix for clustering. Resulting clusters were ranked according to gene size, and only the first ten clusters, with size >100 genes, were considered for subsequent analyses. Enrichment analysis of gene families was performed with ToppFun (https://toppgene.cchmc.org/). Genes coexpressed with Annexins were identified with a correlation test implemented in *R*, using scaled expression values (one-tailed correlation test, *P* < 0.05). Enrichment analysis with Gene Ontology terms and KEGG pathways were performed with the clusterProfiler Bioconductor package.

### Quantitative real time-PCR (q-PCR)

For qPCR analysis, cDNA synthesis was performed using the RevertAid First Strand cDNA Synthesis Kit (Thermo Scientific) and random primers according to manufacturer's protocol. qPCR was carried out in the CFX Connect Real-Time PCR Detection System (BioRad) using Kapa Syber Fast qPCR Mastermix (Kapa Biosystems) in 10 µL final reaction volume. Primer sequences are reported in Supplemental Table S2. Reaction conditions were as follows: one step of 95°C for 3 min, 40 cycles of 95°C for 2 sec of denaturation, and 60°C for 25 sec of annealing and extension, followed by melting curve analysis (from 65°C to 95°C, increment of 0.5°C every 0.5 sec). Ct values were used to calculate the fold change of each gene using the delta/delta Ct method and Glyceraldehyde 3-phosphate dehydrogenase (GAPDH) as reference gene.

### Immunofluorescence

Following treatments (cells were fixed for 15 min in 4% PFA in PBS, quenched (0.38% glycine, 0.24% NH_4_Cl in PBS) and permeabilized with 0.3% Triton X-100 in PBS for 5 min at room temperature. After saturation with 3% goat serum in PBS for 1 h, samples were incubated with primary antibodies diluted in 3% goat serum in PBS overnight at 4°C, washed and incubated with the corresponding secondary antibody (Alexa-conjugated) for 1 h at room temperature. Coverslips were mounted in Mowiol and examined by confocal (Leica SP5) or epifluorescence (Leica CTR6000) microscopy.

### NMJ immunohistochemistry

Upon isoflurane anesthetization, 2-mo-old transgenic C57BL/6 mice of around 20–25 g were locally injected, close to the LAL muscles, with α-LTx (5 µg/kg) diluted in 15 µL of physiological saline buffer (0.9% w/v NaCl in distilled water). Control animals were injected with saline solution. The immunohistochemistry procedure is described in Duregotti et al. (2015).

### Microfluidic devices

Microfluidic chambers were produced using established methods (Park et al. 2006). Polydimethylsiloxane (Dow Corning) inserts were sterilized and fixed to 50 mm glass-bottomed WillCo dishes (IntraCel) using plasma cleaning. The chambers were blocked with 0.8% BSA in PBS overnight at 37°C and then coated with poly-l-ornithine and laminin. Two different configurations of the microfluidic chamber were used: In the first, SC grown in one chamber and H_2_O_2_ added to the opposite compartment, and in the second, MN plated in one compartment and SC in the opposite one.

### Orientation score

Changes in cell orientation were expressed by an orientation score (OS), calculated on the basis of the amplitude of angles (∝) between the main axis of SC processes and the direction of the applied stimulus, represented by the horizontal microfluidic microgrooves. Angles with a coherency value (a parameter to estimate the quality of the measurement) higher than 0.1 were determined using the ImageJ plugin Orientation J (http://bigwww.epfl.ch/demo/orientation/). Measurements were performed on a total of 200 cells and values calculated with the following formula:

$$f\;(x)=1-\sin^{2}(\alpha ).$$

GraphPad Prism software was used for statistical analyses. Quantitative data displayed as histograms are expressed as means ± SD (represented as error bars). Significance was calculated by Student's *t*-test. *P*-values <0.05 were considered significant.

### Puromycilation assay

SC (60%–70% confluence) or cocultures of SC and SCMN were exposed to 50 µM H_2_O_2_ or to 1 nM α-LTx, respectively, for 1 h and then processed as in David et al. (2012) and Yasuda et al. (2013): Briefly, cells were treated with 100 µg/mL cycloheximide at 37°C for 15 min in KRH buffer (NaCl 125 mM, KCl 5 mM, Hepes 25 mM, CaCl_2_ 2 mM, MgSO_4_ 1.2 mM, KH_2_PO_4_ 1.2 mM, and glucose 8 mM, pH 7.4) to block tRNAs within active ribosomes, followed by incubation with 100 µg/mL puromycin at 37°C for 5 min. Samples were then permeabilized in KRH containing 100 µg/mL cycloheximide and 0.0003% digitonin for 2 min on ice. After permeabilization, cells were washed with KRH containing 100 µg/mL cycloheximide and fixed for 15 min with 4% PFA in PBS plus 100 µg/mL cycloheximide, followed by a washing step in PBS. After saturation with 0.1% Triton X-100 in PBS, 5% BSA for 30 min at 37°C, samples were incubated with a primary antibody against puromycin diluted in 5% BSA overnight at 4°C. After extensive washings samples were saturated in 1% BSA in PBS for 15 min and then incubated with the corresponding secondary antibodies for 1 h at room temperature. Additional washing steps with PBS were performed before starting the immunofluorescence protocol as described above.

### Fluorescence in situ hybridization (FISH)

The Probe Designer tool was used to design a set of 48 oligonucleotide probes (Quasar570-conjugated) complementary to the *Rattus norvegicus* Anxa2 transcript (NM_019905.1). Stellaris RNA FISH buffers and probes were purchased by LGC Biosearch Technologies. In situ hybridization was performed on primary SC according to the manufacturer's guidelines for cell cultures. Images were collected as above.

### Western blotting

Following H_2_O_2_ treatment samples were lysed in lysis buffer (Hepes 10 mM, NaCl 150 mM, SDS 1%, EDTA 4 mM, protease inhibitors cocktail [Roche], and phosphatase inhibitor cocktail). Seven to ten micrograms of total lysates from SC were loaded on Precast 4%–12% SDS-polyacrylamide gels (Thermo Fisher Scientific) and transferred onto nitrocellulose paper in a refrigerated chamber. After saturation, membranes were incubated o/n with primary antibody followed by a secondary anti-mouse or anti-rabbit secondary antibody conjugated to HRP. Chemiluminescence was developed with the Luminata Crescendo (Millipore) and emission measured with Uvitec imaging systems. For densitometric quantification, the bands of interest were normalized to the housekeeping protein Hsp90. Band intensities were quantified on the original files with the software Alliance Nine of Uvitec imaging systems. None of the bands reached signal saturation.

### Statistical analysis

qPCR were performed in four biological replicates and 2–3 technical replicates. Results were averaged and the mean value ± SEM is shown and used to calculate the significance by Student's *t*-test (one-tailed unpaired *t*-test; [*] *P* < 0.05, [**] *P* < 0.01, [***] *P* < 0.001).

For western blots, at least three biological replicates were performed. GraphPad Prism software was used for all statistical analyses. Data displayed as histograms are expressed as means ± SD (represented as error bars). Results from each group were averaged and used to calculate descriptive statistics. Significance was calculated by Student's *t*-test (unpaired, two-side). *P*-values <0.05 were considered significant.

## SUPPLEMENTAL MATERIAL

Supplemental material is available for this article.

## Supplementary Material

Supplemental Material
