# Supplementary material for: Hydrogen peroxide is a neuronal alarmin that triggers specific RNAs, local translation of Annexin A2, and cytoskeletal remodeling in Schwann cells
Source: RNA. 2018 Jul;24(7):915–25. doi: 10.1261/rna.064816.117 (PMC6004060; doi:10.1261/rna.064816.117)
Supplement: Supplemental Material [file supp_24_7_915__index.html]

Hydrogen peroxide is a neuronal alarmin that triggers specific RNAs, local translation of Annexin A2, and cytoskeletal remodeling in Schwann cells — Supplemental Material 

# Hydrogen peroxide is a neuronal alarmin that triggers specific RNAs, local translation of Annexin A2, and cytoskeletal remodeling in Schwann cells

## Supplemental Material

- Supplemental\_Figure\_S1.tif
- Supplemental\_Figure\_S2.tif
- Supplemental\_Figure\_S3.tif
- Supplemental\_Figure\_S4.pdf
- Supplemental\_Legends.docx
- Supplemental\_Table\_1.xlsx
- Supplemental\_Table\_2.docx
- Supplemental\_Table\_3.xlsx
