# Supplementary figures and images for: Hydrogen peroxide is a neuronal alarmin that triggers specific RNAs, local translation of Annexin A2, and cytoskeletal remodeling in Schwann cells
Source: RNA. 2018 Jul;24(7):915–25. doi: 10.1261/rna.064816.117 (PMC6004060; doi:10.1261/rna.064816.117)

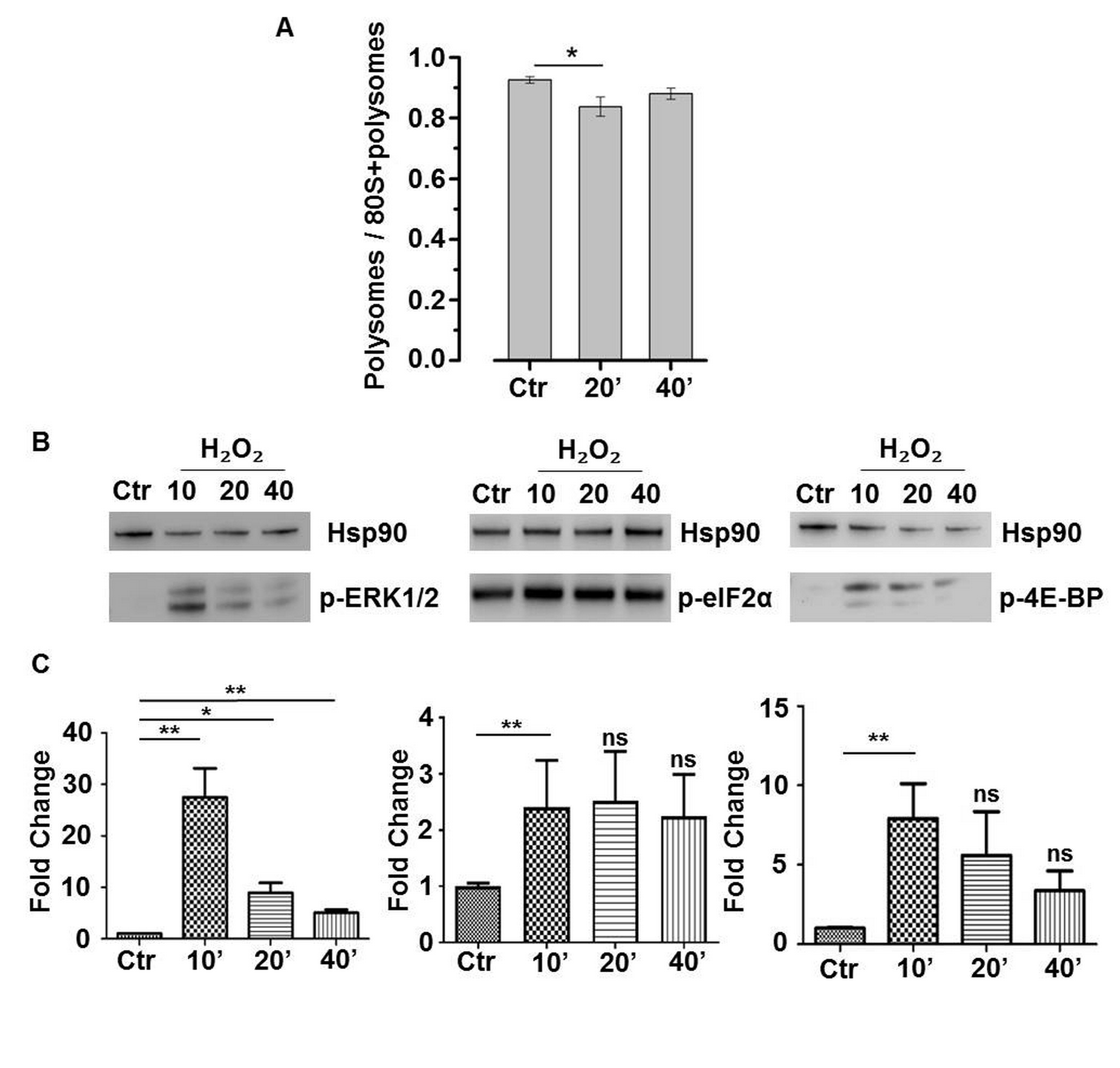

Supplement: Supplemental Material [file supp_064816.117_Supplemental_Figure_S1.tif]

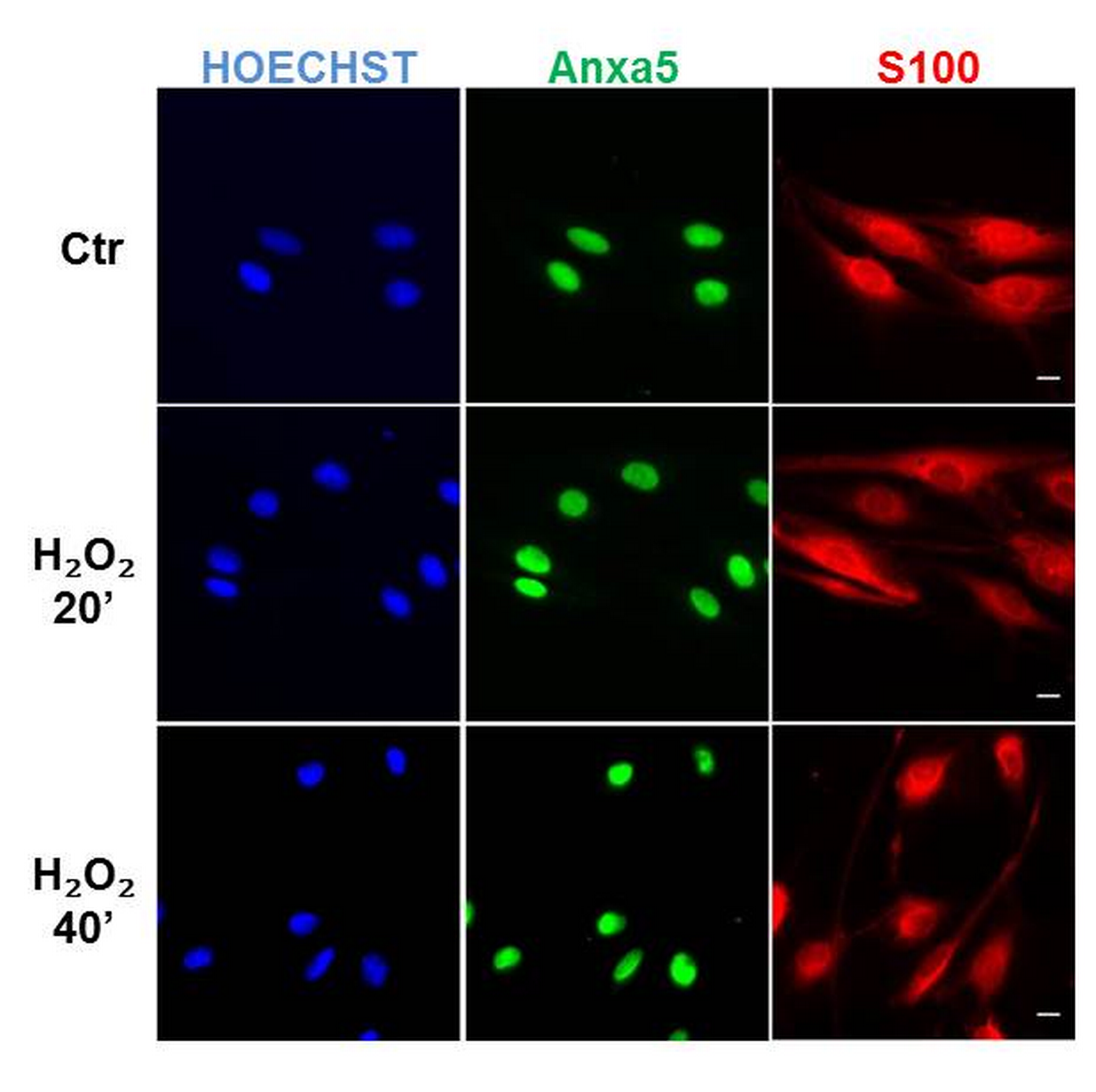

Supplement: Supplemental Material [file supp_064816.117_Supplemental_Figure_S2.tif]

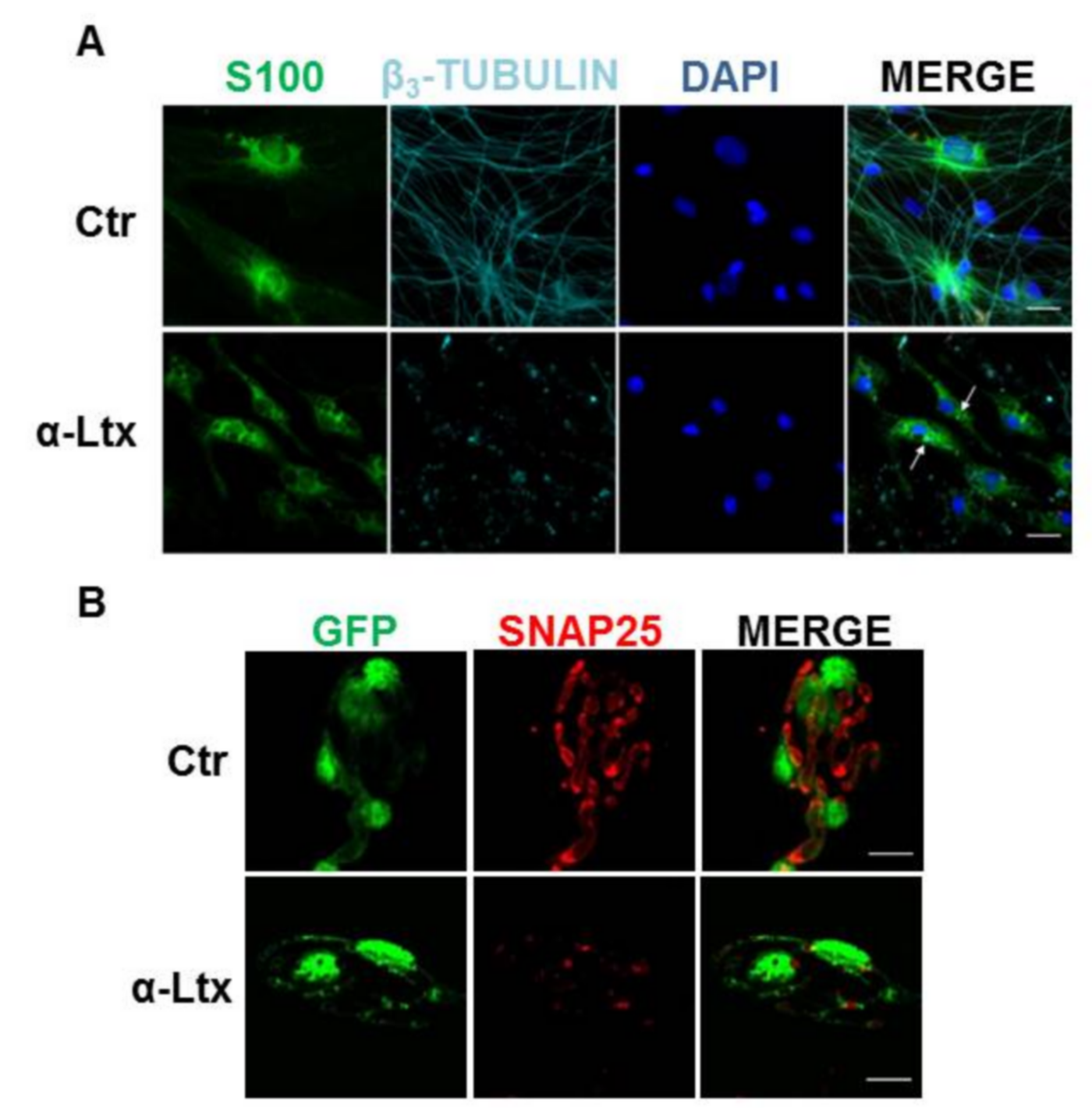

Supplement: Supplemental Material [file supp_064816.117_Supplemental_Figure_S3.tif]

# *Clements et al, 2017*

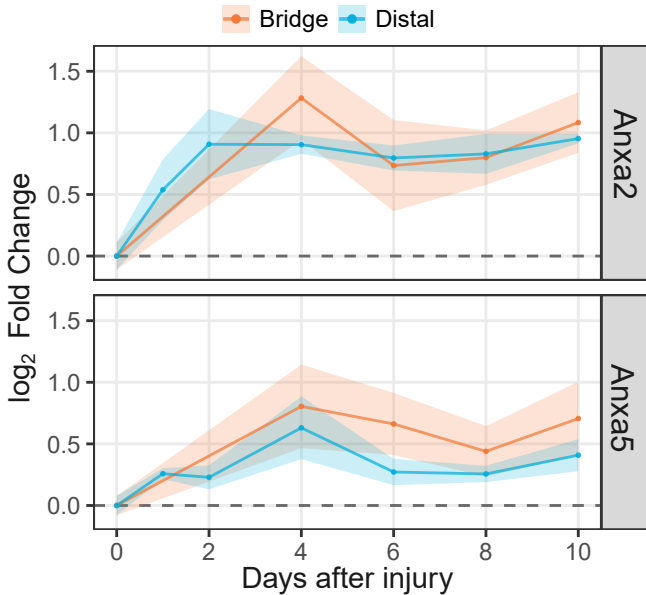

Supplement: Supplemental Material [file supp_064816.117_Supplemental_Figure_S4.pdf]

**Supplementary Table 2:** Primers set used in Real-time PCR experiments


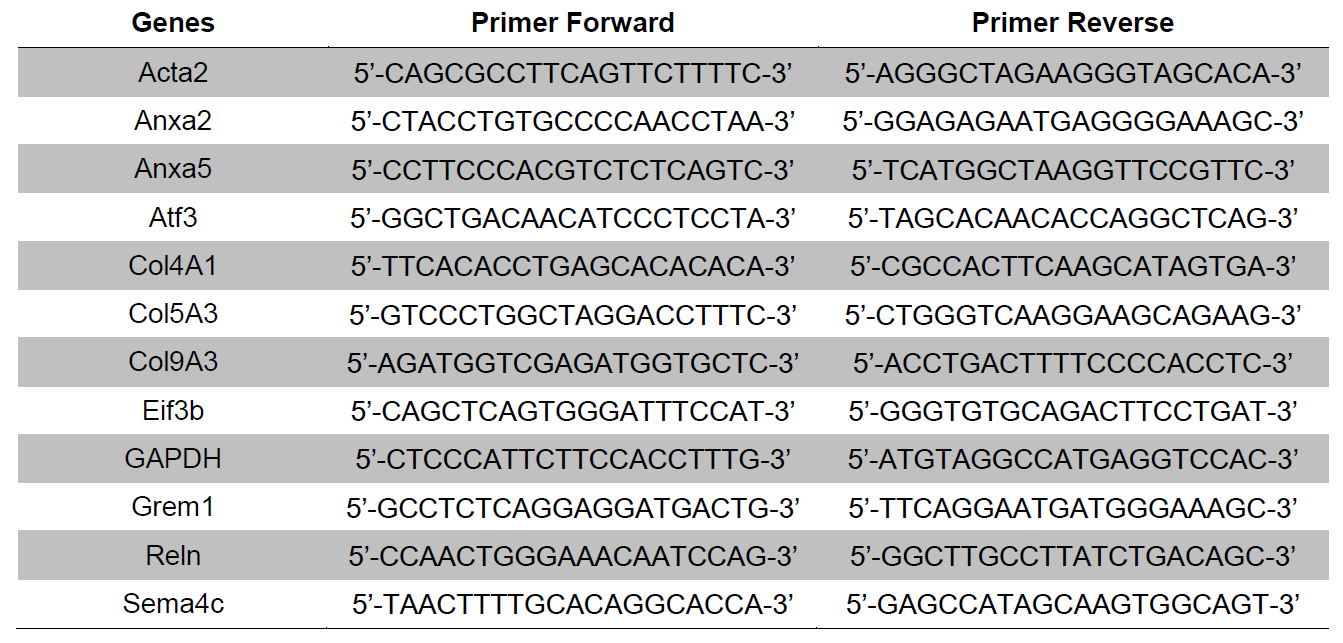

Supplement: Supplemental Material [file supp_064816.117_Supplemental_Table_2.docx]
