# Supplementary material for: Hydrogen peroxide is a neuronal alarmin that triggers specific RNAs, local translation of Annexin A2, and cytoskeletal remodeling in Schwann cells
Source: RNA. 2018 Jul;24(7):915–25. doi: 10.1261/rna.064816.117 (PMC6004060; doi:10.1261/rna.064816.117)
Supplement: Supplemental Material [file supp_064816.117_Supplemental_Legends.docx]

**SUPPLEMENTARY INFORMATION**

**SUPPLEMENTARY FIGURE 1** Translation depression in SC exposed to H_2_O_2_. (a) Global translation defects where calculated using absorbance profiles. The ratio between the absorbance of polysomes and the total absorbance of non-translating 80S ribosomes and polysomes over time is shown. Mean ± sem of 2-7 biological replicates is reported. Significant changes between ctrl and H2O2-treated SC were identified with two-tailed t-test; * P < 0.05. (b) Lysates of SC exposed to H_2_O_2_ 50 μM for 10, 20 and 40 min were probed for phospho-ERK1/2, phospho-eIF2α and phospho-4E-BP. Hsp90 bands represent the internal loading control. Representative western blots are presented. (c) Histograms show quantifications of at least n= 3 blots. * P < 0.05, ** P <0.01, ns= not significant.

**SUPPLEMENTARY FIGURE 2** Anxa5 nuclear localization in SC. Anxa5 staining (*green*) of primary SC (S100 positive, *red*) before and after exposure to 50 μM H_2_O_2_ for 20 (middle panels) and 40 min (bottom panels). Nuclei are stained by Hoechst. Scale bars: 10 μm.

**SUPPLEMENTARY FIGURE 3** Nerve terminal degeneration triggers SC phagocytosis *in vitro* and *in vivo*. (a) Exposure of MN (β_3_-tubulin-positive, *cyan*) to α-LTx (0.1 nM for 4 h) causes neuronal fragmentation and phagocytosis by SC (S100 positive, *green*). Nuclei are stained by Hoechst (*blue*). Scale bars: 10 μm. Arrows point to neuronal debris engulfed in SC phagosomes. (b) α-LTx local injection in *Levatoris auris longus* mice muscles causes axon terminal fragmentation (SNAP25 positive, *red*) (4 h incubation). Neuronal debris are engulfed by PSC (GFP positive, *green*). Scale bar: 10 μm.

**SUPPLEMENTARY FIGURE 4** Time-series gene expression analysis of Anxa2 and Anxa5 in vivo SCs from bridge and distal stumps of transected nerves, compared with SCs from intact nerves (Day 0). Expression values were downloaded from (Clements et al. 2017). Average log2 Fold Change values ± SEM (ribbons) are shown.

**SUPPLEMENTARY TABLE 1:** NGS summary statistics

**SUPPLEMENTARY TABLE 2:** Primer sets used for real-time PCR validation

**SUPPLEMENTARY TABLE 3:** NGS expression and variation data for differentially expressed genes included in clustering analysis
